# Highly Recommended? How Relation-Specific Attachment Styles Bias Customers Willingness to Recommend

**DOI:** 10.3389/fpsyg.2020.01311

**Published:** 2020-06-10

**Authors:** Willem J. M. I. Verbeke, Maarten J. Gijsenberg, Larissa M. E. Hendriks, Jelle T. Bouma, Linda H. Teunter

**Affiliations:** ^1^Erasmus University Rotterdam, Rotterdam, Netherlands; ^2^Department of Marketing, Faculty of Economics and Business, University of Groningen, Groningen, Netherlands; ^3^MetrixLab, Rotterdam, Netherlands; ^4^Customer Insights Center, University of Groningen, Groningen, Netherlands

**Keywords:** relation specific and general attachment styles, willingness to recommend, avoidant attachment style, anxious attachment style, appraisal bias of customer experience

## Abstract

Recently concepts from attachment theory are being applied to business situations. In this paper we focus on how relationship specific (RS) versus general (G) attachment styles affect the willingness-to-recommend (WtR) by customers. Such WtR refers to the likelihood of customers to recommend the services of their service provider to other customers, based on their experiences with the provider. This WtR is often measured by means of the Net Promoter Score (NPS) which is assumed to be a reliable (credible) market signal as it originates from customers themselves and not from the firm. This study provides insights in this issue using data from 798 members of an online panel from the Netherlands, covering four service industries. Customers are surveyed on their RS and G attachment styles, trust in, satisfaction with, and commitment to their service provider, as well as their WtR this provider. Findings emerge from econometric parallel mediation analyses. This study shows that customers’ RS but not the G attachment styles bias their appraisal of trust in, satisfaction with and commitment to the service provider, which in turn affects their WtR. More specifically, across the four service industries, customers scoring higher on RS anxiety and/or avoidance show systematically lower levels of trust in and satisfaction with, and commitment to the firm, ultimately leading to lower WtR. Firms should especially target those customers that score higher on RS avoidance (possibly in combination with higher levels of RS anxiety) as their WtR is strongly biased which might create uncertainty for other customers about the firm’s reputation.

## Introduction

In a decade where customers show lower trust in service providers (Accenture, 2016), customers in their quest for relevant information about the trustworthiness and service quality of the service provider not only study what a service provider herself communicates but, more importantly, customers devote ever more attention to the word-of-mouth activities of other customers about the service provider. In this situation, recommendations by other customers play an important role (e.g., Kiecker and Cowles, 2002; Hennig-Thurau et al., 2004; Goldsmith and Horowitz, 2006; Babić Rosario et al., 2016). When customers are satisfied with the firm, they are willing or even love to recommend the firm (e.g., Hennig-Thurau et al., 2004), ultimately leading to a higher probability that other customers are also purchasing the services (e.g., Boulding and Kirmani, 1993). It consequently should not come as a surprise that there have been multiple calls to account for this willingness-to-recommend (henceforth called WtR) by customers when calculating customer lifetime value and customer equity (e.g., von Wangenheim and Bayón, 2007; Kumar and Reinartz, 2016). Many firms thereby use the net promoter score (henceforth called NPS) as introduced by Reichheld (2003) as a summary statistic of their customers’ WtR to evaluate their service performance, and actively steer on it, with managers being evaluated and rewarded on the firm’s NPS performance.

This study focuses on how WtR is systematically biased by the psychological functioning of the customers’ attachment styles’ working model. Such model consists of the mental representations of the self and others, based on interpersonal experiences (free adopted from Fraley et al., 2011, p. 615). They play a role when appraising the trust in and satisfaction with, as well as commitment to the firm. More concretely, attachment styles reflect psycho-biological hardwired activations of the attachment system shaped by social experience with caretakers in early childhood that carry over in adulthood, thus affecting the way in which people form relationships with other people during adulthood (see next chapter for an explanation). Recently, attachment styles have attracted the attention of business scholars, such as in marketing, because they also affect how customers build relationships with firms (Mende and Bolton, 2011; Mende et al., 2013). Two aspects of this study by Mende and Bolton need mentioning. First, the authors use relationship specific (henceforth called RS) attachment scales and not general (henceforth called G) attachment styles. In line with Fraley et al. (2011) and Gruda and Kafetsios (2020) we investigate the difference between these two operationalizations of attachment styles and explore how they affect the experience and appraisal of relationships in a commercial service environment. Research thus far has suggested that RS compared to G attachment styles are better predictors of how people appraise and develop relationships (e.g., Klohnen et al., 2005; Fraley et al., 2011). Other authors, however, show that G attachment styles are good predictors of competence related appraisals of manager’s leadership effectiveness in an organizational setting (Gruda and Kafetsios, 2020). Second, Mende and Bolton (2011) and Mende et al. (2013) focus on relationship quality which is an abstract concept that researchers use when customers appraise the services of a firm. Here, however, we rather seek to make finer psychological dimensions when customers appraise and develop relationships with firms; specifically, we distinguish between trust in, satisfaction with, and commitment to the firm. These dimensions are conceived as independent dimensions, ranging from quality/performance to relationship dimensions, and allow us to better understand how RS versus G attachment styles relate to these dimensions. Earlier research already has shown that depending on the kind of relationship; e.g., transactional versus relational these three dimensions play a different role in how customers go about these firms (e.g., Garbarino and Johnson, 1999; Athanasopoulou, 2009). To the best of our knowledge this approach, which combines (a) comparing RS to G attachment styles measures and (b) unbundling the relationship concept in sub-dimensions, has not been implemented in a marketing context. Such context, however, provides a powerful environment to foster insights on this issue, as customers not only provide a final appraisal of the performance of the service firm, but also develop relationships with the firm. It thus allows in a unique way for insights relevant to scholars and practitioners alike on the possibly systematically different ways in which attachment styles bias relationship aspects and resulting performance appraisal.

The paper flows as follows. First we briefly discuss how psychologically spoken the working models that characterize how attachment styles operate within individuals and present a model showing how customers’ RS versus G attachment styles bias customers’ judgment of the firm in terms of trust in, satisfaction with, and commitment to the service firm, which in turn affects the customers’ WtR. We subsequently present our data, discuss the applied methodology, and present research results. We end by discussing consequences for theory, making recommendations to managers and discussing some limitations of our work.

## Background

### A Psycho-Biological Explanation of Attachment Styles

Interest in attachment styles emerged in the second half of the former century when researchers noticed that human beings are “born with an innate psycho-biological system that motivates them to seek proximity to significant others (attachment figures) in times of need as a way of protecting them from threats and alleviating distress” (Bowlby, 1973; Ein-Dor et al., 2010, p. 124; Ein-Dor and Tal, 2012). Although a matter of discussion, the quality of the child-caretakers interaction during that time period has lasting or imprinting impacts on the way humans later in life appraise the trustworthiness of others, experience satisfaction in life and form relationships with other attachment figures (Mikulincer and Shaver, 2007). Attachment figures/targets include close relationships, friends and colleagues, but also extend to compensatory abstract figures/targets such as religion (Granqvist et al., 2010) but also brands (Batra et al., 2012) and service firms (Mende and Bolton, 2011).

The attachment system actually operates as a goal-directed homeostatic system that allows the child to experience safety and quiescence when it detects danger or stress occurs. Over time it cements into the brain as working model which encodes expectations of care and allows for mental simulation and prediction of likely outcomes of various attachment behaviors (Vrticka and Vuilleumier, 2012, p. 2). Depending on the actual experiences as a child with the reactions of the caretakers, different attachments styles – which differ in the way the working model operates – will develop. These styles then bias the way people experience and appraise their social environment and how they develop relationships with attachment figures/targets (e.g., Waters et al., 2002). Because of the heterogeneity of the just mentioned attachment targets, whether these attachment styles operate invariably across a wide range of relational contexts hence they function as a trait (G attachment styles) or whether they vary across different contexts (RS attachment styles) and show low to modest correlations among each other (Overall et al., 2003; Klohnen et al., 2005; Fraley et al., 2011) is a general point of discussion.

The largest group of people develop a so-called *secure* attachment style, which is perceived as the default attachment system modus. These people have experienced consistent caring efforts from their caretakers when in need or stress. These people experience their social environment as less threatening, allowing them to learn and gauge whether a social environment is safe or not, explore their social environment when safe but also turn back to attachment figures/targets in case of need. As a consequence, they evaluate their attachment figures/targets (like friends) as trustworthy and fully enjoy the pleasures which come with companionship, and therefore want to seek proximity with others or form new attachments or relationships with e.g., friends (see Mikulincer and Shaver, 2007). About 65% of the population is estimated to be securely attached (Van IJzendoorn et al., 2006; Bakermans-Kranenburg and van IJzendoorn, 2009).

However, the consistent nurturing behavior by caretakers does not always happen and is open to variation. This can result in children showing a biased appraisal of and desire to attach themselves to attachment figures (Mikulincer and Shaver, 2007; Diamond, 2015). This, in turn, is reflected in higher levels of *anxiety*, and is shown by hyper-activation of the attachment system (Van IJzendoorn et al., 2006). These people’s attention is biased toward detection of threats in their social environment and consequently they fear rejection from attachment figures. Paradoxically, this amplifies their wanting for proximity which then only aggravates fear for rejection. Note the complexity by which their working model evokes push (seeking proximity) and pull (fear for rejection) toward attachment figures (Vrticka and Vuilleumier, 2012). Besides deviating from a secure attachment style by showing a higher degree of anxiety, people can also deviate by showing a higher degree of *avoidance* (Van IJzendoorn et al., 2006). People showing higher levels of avoidance are also biased to attend to signals of negative valence but due to the insensitivity of their caretakers early in life they have learned to expect less or even no proximity from their attachment figures in case of need. As a consequence, they value much less (if at all) close friendships or relationships which normally would reduce their stress or anxiety hence their low desire to develop relationships. As they remain more independent and cannot really gain quiescence with others, their appraisal of other people’s trust and enjoyment of pleasure that comes with proximity is biased, resulting in lower trust in and lower satisfaction with attachment figures. Consequently, their working model almost pushes them away from attachment figures, and the way they build social networks is largely transactional and opportunistic (e.g., Mikulincer and Shaver, 2007).

Finally, for certain people, child-time experiences result in higher levels of both anxiety and of avoidance. When separated from attachment figures, the threats they detect are so overwhelming that these people constantly worry about the trustworthiness of their attachment figures. This, in turn, intensifies their desire for help, yet this desire is also colored with fear. Concretely, and compared to people showing only higher levels of anxiety, it is not so much fear of being rejected but fear of being harmed by the attachment figures. Even when primed with positive stimuli, these people respond in confused ways to these social stimuli (Bateman and Fonagy, 2005).

While attachment style was originally regarded as an overall personal trait that was the same regardless of the type of relationship, recent research has argued that an individual’s attachment style may vary depending on the specific relationship with the attachment figures or targets, thereby deviating from the just described general “psycho-biological” attachment style of an individual (e.g., Bartz and Lydon, 2004). Given the more specific nature of the relationship-specific (RS) attachment style regarding the relationship with attachment figures/targets, it is not surprising that RS attachment style has been shown to have stronger predictive value for the relationship at hand, compared to general (G) attachment style which is referentially ambiguous (Fraley et al., 2011, p. 615; Klohnen et al., 2005). In this paper we will adopt this RS attachment style perspective and compare it with the G attachment style in how it will affect people’s relationship experiences, and ultimately appraisal, in a commercial service environment where both cognitive (performance) and emotional (e.g., satisfaction) aspects play an important role.

### A Theoretical Model of the Customer-Firm Interaction Process

**FIGURE 1:**
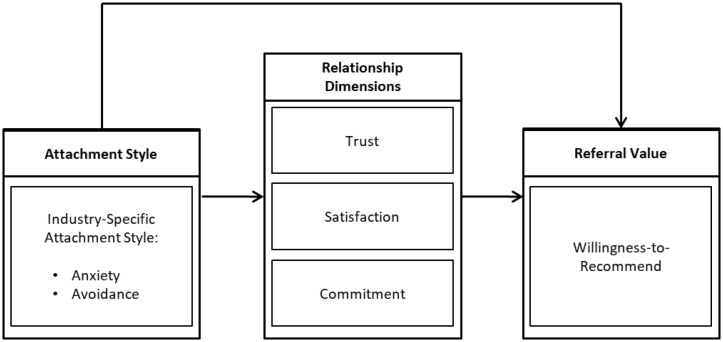
The customer-firm interaction process model.

Our model as depicted in Figure 1 starts from the premise that customers’ RS versus G attachment style biases both their appraisal of the service provider and their commitment to the service provider, as reflected in customers’ trust in, satisfaction with, and commitment to the firm, which we gather under the relationship aspects denominator.

Given that the interaction between firms and customers entails a risk (e.g., financial risks or opportunity costs) and uncertainty (e.g., “is this firm truly concerned with my problems?”), customers will appraise whether business partners are reliable and predictable. They hope that the business partners are usually concerned with their needs and can be counted on in times of need. Trust reflects the appraisal of others as dependable and predictable, and the belief that a partner is concerned with one’s own needs in case of stress or separation (Mikulincer, 1998). As such, trust is necessary for successful customer relationship building (Berry, 1995), to reduce attention focus on relational risks (Nooteboom et al., 1997) and thus to provide psychological comfort (Rempel et al., 1985).

The second aspect we include in our model, is the customers’ satisfaction with the firm (e.g., Mende et al., 2013). Customer satisfaction can be regarded as the outcome of the expectancy-disconfirmation paradigm with satisfaction as the result of a customer’s comparison between perceived and expected service performance over time (e.g., Churchill and Surprenant, 1982; Rust et al., 1999). Furthermore, satisfaction also implies an emotional state (such as pleasure and liking) that occurs as a result of customer’s interactions with the firm over time (Crosby et al., 1990; Anderson et al., 1994; Gijsenberg et al., 2015). This definition of satisfaction with the firm consequently takes a holistic perspective and does not look at the evaluation of one specific service employee or one specific service encounter in time. As such, it looks at the overall evaluation of the whole experience with the firm, built over all service encounters from the past and present.

Third, commitment, is the positive emotional (e.g., seeking again or wanting enjoyment with interaction with the firm) or psychological state of attachment to a brand or firm that develops and differs from merely habitual repeat-purchase behavior (Beatty and Kahle, 1988). Commitment to a firm thus measures customers’ attachment to a brand/firm and implies an enduring desire to continue a relationship because it provides comfort (Morgan and Hunt, 1994). Commitment, moreover, is the most prominent perception representing the strength of the relationship (Moorman et al., 1992; Morgan and Hunt, 1994).

The final part in the model focusses on the referral aspect of the relationship evaluation focusing on customers’ WtR. Customer-recommendation behaviors have become an increasingly important area of study about the behavior of customers (e.g., Anderson, 1998; Verhoef et al., 2002). This results from two yet to be validated “assumptions”: (1) When people are willing to tell how satisfied they are to others they express the sincerity of their beliefs; (2) Recommendation behavior is the consumer post-purchase phenomenon that produces the greatest benefit for supplier firms (e.g., Johnson and Selnes, 2004; Brown et al., 2005). All three customer-firm relationship aspects included in the model – trust, satisfaction, and commitment, – have been shown to be important drivers of customers’ WtR (De Matos and Rossi, 2008). While important across many industries, customer recommendations are even more so in service industries, as often services can only be evaluated after a longer period of being in a service relationship (which is also called experienced utility; Kahneman et al., 1997). Earning recommendations from customers can be a powerful force in augmenting a company’s marketing efforts for two reasons: (1) trust in service providers has been at an all-time low (Accenture, 2016), and (2) customers have become connected customers. The latter might amplify both negative and positive market signals (Kirby and Mardsen, 2005) where for example the volume of positive electronic WtR has a positive impact on firms’ sales (Babić Rosario et al., 2016).

### RS and G Attachment Styles and WtR

While, based on the aforementioned literature, we expect RS attachment styles to show considerably stronger relationships with the relationship dimensions compared to the G attachment styles, we still expect the G attachment styles to also show significant effects on the different relationship dimensions. As a consequence, in our proposed hypotheses we make similar conjectures for both the RS and G attachment styles.

Despite the fact that buying services from firms implies risk (e.g., financial risk in engaging with a firm over a long period due to lock-in long-term contracts) we might expect customers scoring low on both (RS and G) anxiety and avoidance due to the healthy functioning of their working model (1) to experience trust in the brand or firm (e.g., feeling that the firm is concerned with their welfare), (2) to experience satisfaction with the firm as they can without a bias consider the ratio of positive and negative sides of the service delivered over time and make evaluations accordingly, and (3) to show high commitment to the firm (e.g., being attached to it or enjoy being a customer of that firm). Most important for a customer relationship with a firm context, however, these people also both can and are likely to share their experiences about the service as they (1) have the cognitive resources to make judgments about their social environment and (2), due to their communal motivations, will fully provide consumer platform assistance around the brand and show more concern that other customers are also well-informed about the firm (Hennig-Thurau et al., 2004). This ability will hence be reflected in their WtR, where their higher levels of trust, satisfaction and commitment will also lead to *actual* positive recommendations (e.g., De Matos and Rossi, 2008).

As Mende and Bolton (2011) show, customers scoring higher on (RS and G) anxiety tend to have a biased appraisal of their *overall* relationship quality. This overall picture, however, may forego subtle differences when it comes to the different aspects of relationship quality. These people might well fear more rejection from relationships, but actually they have a higher need for proximity and thus they might show higher commitment. However, even though they are more committed, they might be more sensitive or better critical about the sincerity by which the company provides them trust or critical about the quality the company provides them.

Despite the fact that customers scoring higher on (RS and G) avoidance actually still buy the services, such customers show lower trust in the firm, do not attain the same levels of satisfaction neither do they really want to remain committed to a firm, which reflect the push direction of their working model (Mende and Bolton, 2011). First, they remain more sensitive to adverse messages communicated to them by the firm and are less likely to trust the firm despite trust-increasing or assuring messages from the firm. Such negative feelings and reduced trust may even be exacerbated by their lower ability to cope with stress caused by adverse messages (e.g., Verbeke et al., 2018). Next, as these customers are less likely to enjoy pleasures, we can expect them to have lower degrees of customer satisfaction despite all efforts from the firm. Finally, as their need to bond with others is lower, we expect them to have lower degrees of customer commitment. As a consequence, they will respond less (or not) to standard firms’ efforts, resulting in lower WtR. Just like with customers scoring higher on RS and G anxiety, the WtR of customers scoring higher on (RS and G) avoidance is not reliable as a signal of the service experience.

Contrary to Mende et al. (2013) we do not expect that higher levels of (RS and G) anxiety and/or avoidance will negatively bias the customers’ appraisals of trust, satisfaction, and commitment and ultimately their WtR in a similar way. People showing higher levels of only (RS and G) anxiety still want to have or crave for a close relationship, and will closely monitor the interaction, but these judgments remain biased. This, however, is likely to have a smaller impact than the increasing tendency of people showing higher levels of only (RS and G) avoidance (who are more sensitive to negative signals while at the same time keeping a distance to the firm) to not trust the firm, neither enjoy its services, nor show any commitment to the firm.

Summarizing the reasoning provided above, we formulate a concise set of hypotheses. First, based upon the discussion on the different RS and G attachment styles, we state that:

**H1a:** RS anxiety has a negative effect on a customer’s WtR.
**H1b:** RS avoidance has a negative effect on a customer’s WtR.
**H1c:** RS avoidance has a stronger effect on WtR than RS anxiety.
**H1d:** G anxiety has a negative effect on a customer’s WtR.
**H1e:** G avoidance has a negative effect on a customer’s WtR.
**H1f:** G avoidance has a stronger effect on WtR than G anxiety.

Next we assume that, due to the different push-pull dynamics which the working models of the RS and G attachment system evoke, there will be differences in how RS and G attachment styles are related to the three dimensions of customer relationships. Hence we expect that Mende et al. (2013) will not be replicated.

**H2a:** RS anxiety has a negative effect on a customer’s trust in the firm.
**H2b:** RS avoidance has a negative effect on a customer’s trust in the firm.
**H2c:** RS avoidance has a stronger effect on trust than RS anxiety.
**H2d:** G anxiety has a negative effect on a customer’s trust in the firm.
**H2e:** G avoidance has a negative effect on a customer’s trust in the firm.
**H2f:** G avoidance has a stronger effect on trust than G anxiety.
**H3a:** RS anxiety has a negative effect on a customer’s satisfaction with the firm.
**H3b:** RS avoidance has a negative effect on a customer’s satisfaction with the firm.
**H3c:** RS avoidance has a stronger effect on satisfaction than RS anxiety.
**H3d:** G anxiety has a negative effect on a customer’s satisfaction with the firm.
**H3e:** G avoidance has a negative effect on a customer’s satisfaction with the firm.
**H3f:** G avoidance has a stronger effect on satisfaction than G anxiety.
**H4a:** RS anxiety has a negative effect on a customer’s commitment to the firm.
**H4b:** RS avoidance has a negative effect on a customer’s commitment to the firm.
**H4c:** RS avoidance has a stronger effect on commitment than RS anxiety.
**H4d:** G anxiety has a negative effect on a customer’s commitment to the firm.
**H4e:** G avoidance has a negative effect on a customer’s commitment to the firm.
**H4f:** G avoidance has a stronger effect on commitment than G anxiety.

Finally, integrating the attachment styles literature, the work by Mende and Bolton (2011) and the word of mouth literature (e.g., De Matos and Rossi, 2008), we would hypothesize that the effect of a customer’s RS and G attachment style on her WtR is fully mediated, i.e., indirect through its effect on trust, commitment and satisfaction. However, Mende et al. (2013) also show remaining direct effects on their outcome variables after accounting for trust, commitment and satisfaction. Given these mixed arguments, we state the following hypotheses:

**H5a:** The effect of a customer’s RS attachment style on her WtR is fully mediated by its effect on her trust in, satisfaction with, and commitment to the firm, i.e., it only has an indirect effect on WtR.
**H5b:** The effect of a customer’s RS attachment style on her WtR is partially mediated by its effect on her trust in, satisfaction with, and commitment to the firm, i.e., it has a direct and indirect effect on WtR.
**H6a:** The effect of a customer’s G attachment style on her WtR is fully mediated by its effect on her trust in, satisfaction with, and commitment to the firm, i.e., it only has an indirect effect on WtR.
**H6b:** The effect of a customer’s G attachment style on her WtR is partially mediated by its effect on her trust in, satisfaction with, and commitment to the firm, i.e., it has a direct and indirect effect on WtR.

## Data and Measurement

### Data

For this research, we had access to data from the online panel of MetrixLab, a marketing research company in the Netherlands. This panel is a representative sample of the Dutch population. All data obtained from this panel are subject to informed consent by the panel members: when people sign up for panel membership, they are informed of and agree with the fact that data collected through this panel can be used for research purposes by MetrixLab and its partners.

The data used in this research were collected by MetrixLab for internal research purposes other than the current study. As such, the data are of a similar nature as data obtained from other panel data service providers like Kantar, GfK, or Nielsen. This type of data is commonly used in academic marketing research to trace effects of marketing actions on for instance mindset metric and sales (see, e.g., Gijsenberg, 2017 for an example). The current data can consequently be considered proprietary secondary data^1^.

Panel members received an online survey which included the measurement scales of G attachment style, RS attachment style, trust, customer satisfaction, commitment, and WtR described below. In total 798 respondents (44.6% male and 55.4% female) filled out the survey. The average age of the participants was 46.6 (*SD* = 14.3) years.

The included data cover four service industries, namely banking, insurance, energy, and telecom. The interaction between the firm and the customer is extremely relevant in such service industries. The inclusion of multiple industries allows us to investigate to what extent the effect of attachment style on WtR follows generalizable patterns, thus overcoming the idiosyncrasies of a single industry.

Each respondent was asked to provide the name of her main bank, insurance company, energy supplier and telecom firm. This served as the selection question, as respondents were not always able to provide the name of all four service providers. Based on the answers, two industries were randomly assigned to each respondent^2^, making sure to assign only an industry in which they knew their service provider. For the 798 respondents participating in the survey, this resulted in 425 evaluations of banks, 368 of insurance companies, 397 of energy suppliers, and 403 of telecommunication firms.

### Measurement

The conceptual framework depicted in Figure 1 displays a set of concepts relating to RS and G attachment styles, relationship dimensions, and WtR. We operationalize these concepts using established measures that are well-grounded in previous research, thus also facilitating the comparison of our findings with extant literature. Here we provide a concise overview of the measures used; we refer to Supplementary Appendix A for a detailed description of the measures.

We measure G attachment style using the scale developed by Verbeke et al. (2013). This scale consists of 11 items, of which six items measure the anxiety dimension and five items measure the avoidance dimension. To measure RS attachment style, we use the scale developed by Mende and Bolton (2011), who based their instrument on the Experiences in Close Relationships (ECR) scale (Brennan et al., 1998) and its revised version, the ECR-R (Fraley et al., 2000). Mende and Bolton (2011) conducted three scale development studies in various service contexts. They came up with four items for the two dimensions anxiety and avoidance, and demonstrated high reliability and validity of the instrument, which was confirmed in the research on industry-specific attachment styles by Mende et al. (2013).

Three concepts cover an equal number of dimensions of the relationship: trust, satisfaction, and commitment. We measure trust and commitment with three-item scales that were also used in the studies about attachment styles by Mende and Bolton (2011) and Mende et al. (2013). For trust, we use the scale developed by Doney and Cannon (1997) and for commitment items developed by Gruen et al. (2000) and Coulter et al. (2003). Customer satisfaction, in turn, is measured by only one item. Following Aaker et al. (2004) and Mende et al. (2013), we measure customer satisfaction with the statement “I am satisfied with [firm X]” on a five-point Likert-scale, ranging from 1 = “I fully disagree” to 5 = “I fully agree.” As such, this is a holistic measure of satisfaction with the full experience with the service provider.

To measure WtR, we follow Reichheld (2003) who showed that this can be addressed by the question “How likely are you to recommend [firm X] to a friend, relative or colleague on a scale from 0 to 10?” whereby 0 = “extremely unlikely” and 10 = “extremely likely.”^3^

Before proceeding with the analysis, certain items of the multi-item scales are reverse coded to ensure internal consistency. We subsequently tested for the reliability of the multi-item scales using Cronbach’s Alpha. Table 1 reports the results of these tests. All values are well beyond 0.7, showing the reliability of our scales. Single-item scales were not included in this table. We then averaged over the different items of each scale and include these average values in our analyses. For both the RS and G attachment styles, this approach implies one value for the anxiety dimension and one value for the avoidance dimension.

**TABLE 1:** Reliability tests of the scales: Cronbach’s alpha.

**General (G) attachment style dimensions**				
Anxiety (6)	0.81			
Avoidance (5)	0.76			

	**Banking**	**Insurance**	**Energy**	**Telecom**

**Relationship-specific (RS) attachment style dimensions**				
Anxiety (4)	0.85	0.86	0.84	0.83
Avoidance (4)	0.81	0.80	0.81	0.79
**Relationship aspects**				
Trust (3)	0.85	0.82	0.80	0.79
Commitment (3)	0.89	0.86	0.87	0.88

*Numbers between brackets represent the number of included items in the scale.*

### Preliminary Insights: Attachment Dimensions Correlation

Previous literature has argued that a person’s RS and G attachment styles not necessarily correlate (Mikulincer and Shaver, 2007), and has shown that the former might be specific activations of the working models which are more concrete and as a consequence also a better predictor of the outcomes of a relationship (Overall et al., 2003; Klohnen et al., 2005). We therefore expect correlations between both levels of attachment style to be moderate at best. Our data confirm this expectation. Respondents’ G anxiety scores correlated significantly with the RS anxiety scores in all industries (*p* < 0.01), but the correlations were low: 0.25, 0.25, 0.32, and 0.27 for banking, insurance, energy and telecom, respectively. This observation corresponds with findings of Fraley et al. (2011). Respondents’ G avoidance scores only correlated significantly with the insurance RS avoidance score, but here as well, the correlation was low: 0.14 (*p* < 0.01). Thus, correlations between the RS and G attachment styles are low or absent. The G attachment styles thus probably measures a trait-like attachment style while the RS is sensitive to attachment targets, specifically industries or contexts (e.g., Fraley et al., 2011). Instead of interpreting the RS attachment style as an extension of the G attachment style, we have to interpret them as separate constructs as already proposed by Overall et al. (2003) and Fraley et al. (2011) who note that relationship context matters (p. 1491). A detailed overview of all correlations between the included constructs is provided in the Supplementary Appendix.

## Methodology

The conceptual framework in Figure 1 shows how RS and G attachment styles affect WtR. This influence, however, can be indirect through their effect on trust, satisfaction and commitment, a situation of mediation. Our mediation analysis thus proceeds in four steps. Each of these steps is carried out twice: once for the RS attachment styles, once for the G attachment styles.

First, we establish whether and to what extent RS and G attachment styles have an effect on WtR, without taking into account any possible indirect effects. We therefore regress respondents’ WtR on their (RS and G) anxiety and avoidance. We also control for age and gender of the individual customer. For respondent j in industry k, the resulting equation thus becomes:

$$W\,t\,R_{j\,k}=\alpha_{k,0}+\alpha_{k,1}\,A\,n\,x\,i\,e\,t\,y_{j\,i}+\alpha_{k,2}\,A\,v\,o\,i\,d\,a\,n\,c\,e_{j\,i}+\alpha_{k,3}\,A\,g\,e_{j}$$

$$+\alpha_{k,4}\,G\,e\,n\,d\,e\,r_{j}+\omega_{j\,k}$$

where i refers to the respondent’s RS attachment style in industry k or its G attachment style. As we use RS and G attachment styles, the same respondent is allowed to have different attachment styles in different industries.

In a second step, following Mende and Bolton (2011), we determine how RS and G attachment styles affect the different relationship dimensions. We do so by regressing trust, satisfaction, and commitment on the (RS and G) anxiety and avoidance, thereby controlling for age and gender of the individual respondent. For respondent j in industry k, the resulting equations thus become:

$$T\,r\,u\,s\,t_{j\,k}=\beta_{k,0}^{T}+\beta_{k,1}^{T}\,A\,n\,x\,i\,e\,t\,y_{j\,i}+\beta_{k,2}^{T}\,A\,v\,o\,i\,d\,a\,n\,c\,e_{j\,i}+\beta_{k,3}^{T}\,A\,g\,e_{j}$$

$$+\beta_{k,4}^{T}\,G\,e\,n\,d\,e\,r_{j}+\varepsilon_{j\,k}^{T}$$

$$S\,a\,t\,i\,s_{j\,k}=\beta_{k,0}^{S}+\beta_{k,1}^{S}\,A\,n\,x\,i\,e\,t\,y_{j\,i}+\beta_{k,2}^{S}\,A\,v\,o\,i\,d\,a\,n\,c\,e_{j\,i}+\beta_{k,3}^{S}\,A\,g\,e_{j}$$

$$+\beta_{k,4}^{S}\,G\,e\,n\,d\,e\,r_{j}+\varepsilon_{j\,k}^{S}$$

$$C\,o\,m\,m\,i\,t_{j\,k}=\beta_{k,0}^{C}+\beta_{k,1}^{C}\,A\,n\,x\,i\,e\,t\,y_{j\,i}+\beta_{k,2}^{C}\,A\,v\,o\,i\,d\,a\,n\,c\,e_{j\,i}+\beta_{k,3}^{C}\,A\,g\,e_{j}$$

$$+\beta_{k,4}^{C}\,G\,e\,n\,d\,e\,r_{j}+\varepsilon_{j\,k}^{C}$$

where i refers to the respondent’s RS attachment style in industry k or its G attachment style.

Third, we determine how trust, satisfaction and commitment affect WtR, thereby regressing respondents’ WtR on (1) trust, (2) satisfaction, (3) commitment, and (4) (RS and G) anxiety and avoidance, and control for age and gender of the individual respondent. For respondent j in industry k, the resulting equation thus becomes

$$W\,t\,R_{j\,k}=\gamma_{k,0}+\gamma_{k,1}\,A\,n\,x\,i\,e\,t\,y_{j\,i}+\gamma_{k,2}\,A\,v\,o\,i\,d\,a\,n\,c\,e_{j\,i}+\gamma_{k,3}\,T\,r\,u\,s\,t_{j\,k}$$

$$\;\;+\gamma_{k,4}\,S\,a\,t\,i\,s_{j\,k}+\gamma_{k,5}\,C\,o\,m\,m\,i\,t_{j\,k}+\gamma_{k,6}\,A\,g\,e_{j}$$

$$\;\;+\gamma_{k,7}\,G\,e\,n\,d\,e\,r_{j}+\upsilon_{j\,k}$$

In a fourth and final step, we quantify the total indirect effects of (RS and G) anxiety and avoidance on WtR through trust, satisfaction, and commitment. These indirect effects are summarized in Table 2.

**TABLE 2:** Overview of indirect effects of attachment style dimensions on WtR.

	**Through trust**	**Through satisfaction**	**Through commitment**
Anxiety	$\beta_{k,1}^{T}\,\text{*}\,\gamma_{k,3}$	$\beta_{k,1}^{S}\,\text{*}\,\gamma_{k,4}$	$\beta_{k,1}^{C}\,\text{*}\,\gamma_{k,5}$
Avoidance	$\beta_{k,2}^{T}\,\text{*}\,\gamma_{k,3}$	$\beta_{k,2}^{S}\,\text{*}\,\gamma_{k,4}$	$\beta_{k,2}^{C}\,\text{*}\,\gamma_{k,5}$

We calculate the total indirect effects and their confidence intervals using the multiple mediation bootstrap approach proposed by Preacher and Hayes (2008). We thereby use bias-corrected percentile confidence intervals, and set the confidence level to 95. If zero is not contained in the confidence interval, the indirect effect is considered significant. Insights are based on 50,000 bootstrap samples. We repeat steps 1–4 for all four service industries.

## Results

### Effects of RS and G Attachment Styles on WtR

We first provide insights into the extent to which RS and G attachment styles affect WtR by means of the results of the regression analysis described in the first step of our methodology. Table 3 shows the results of this analysis for the effects of both RS and G attachment styles.

**TABLE 3:** Effects of attachment style on WtR.

	**Banking**	**Insurance**	**Energy**	**Telecom**
**Relationship-specific (RS) attachment style**				
Constant	13.01**	12.13**	12.15**	13.20**
Anxiety	−0.82**	−0.81**	−0.73**	−0.90**
Avoidance	−1.41**	−1.23**	−1.23**	−1.46**
Age	–0.00	0.00	0.00	0.00
Gender	–0.10	–0.13	–0.09	0.27

*R*^2^	0.38	0.30	0.24	0.31
*F*-value	62.82**	38.55**	30.94**	44.72**
Max VIF	1.07	1.09	1.090	1.11
BIC	3.913	3.900	4.008	4.069

**General (G) attachment style**				
Constant	7.19**	7.63**	6.75**	7.16**
Anxiety	–0.12	0.12	0.17	–0.05
Avoidance	0.03	−0.54**	–0.22	–0.14
Age	–0.00	0.01	0.00	0.00
Gender	–0.03	–0.22	–0.12	0.18

*R*^2^	0.00	0.04	0.01	0.00
*F*-value	0.30	4.10**	0.61	0.44
Max VIF	1.48	1.50	1.62	1.60
BIC	4.381	4.211	4.276	4.436

*p < 0.05, **p < 0.01.

RS attachment style shows good explanatory power, with *R*^2^ values ranging between 0.24 (energy) and 0.38 (banking). Both general patterns and significance levels are consistent across industries. Respondents scoring higher on anxiety systematically show lower WtR. More importantly, however, avoidance shows a negative effect which is about 1.5 times the size of the effect of anxiety. All individual-industry differences are significant (*p* < 0.01, except insurance: *p* < 0.05). Altogether, these results show strong support for H1a, H1b, and H1c. Interestingly, age and gender do not have a significant impact on respondents’ WtR.

G attachment style, in turn, shows very limited explanatory power, with *R*^2^ values hardly above zero and *F*-values being insignificant, except for the insurance industry. We consequently cannot claim true support for H1d, H1e, and H1f. This superiority of RS attachment style in the analysis of customers’ relations with firms thus confirms earlier observations by Klohnen et al. (2005) as well as Fraley et al. (2011) on the stronger predictive value of relationship-specific attachment style in a focal relationship because working models are activated depending on current goals, emotions and past experience (Overall et al., 2003, p. 1491).

### Direct and Indirect Effects of RS Attachment Style on WtR

In our conceptual framework, we hypothesized that the effects of RS and G attachment styles on WtR were indirect through (mediated by) the three relationship dimensions of trust, commitment, and satisfaction. We tested for this indirect effect, thereby also analyzing whether any direct effects remain after accounting for the indirect effects. If this were the case, partial mediation would occur; if no remaining direct effects are present, perfect or full mediation would occur (James and Brett, 1984). As we have shown G attachment style to be a bad predictor of WtR, we hereby limit the mediation analysis to the RS attachment style. Table 4 reports the results of the analysis.

**TABLE 4:** Mediation analysis of relationship-specific attachment style dimensions on WtR.

		**Banking**	**Insurance**	**Energy**	**Telecom**
**Effect on trust**					
$\beta_{k,1}^{T}$	Anxiety	−0.38**	−0.38*	−0.31**	−0.33**
$\beta_{k,2}^{T}$	Avoidance	−0.61**	−0.56**	−0.51**	−0.61**
**Effect on satisfaction**					
$\beta_{k,1}^{S}$	Anxiety	−0.45**	−0.44**	−0.38**	−0.46**
$\beta_{k,2}^{S}$	Avoidance	−0.52**	−0.45**	−0.51**	−0.54**
**Effect on commitment**					
$\beta_{k,1}^{C}$	Anxiety	−0.29**	−0.27**	−0.21**	−0.26**
$\beta_{k,2}^{C}$	Avoidance	−0.75**	−0.72**	−0.80**	−0.76**
**Direct effect on WtR**					
γ_*k,3*_	Trust	0.24	0.68**	0.62**	0.18
γ_*k,4*_	Satisfaction	0.82**	0.73**	0.74**	0.96**
γ_*k,5*_	Commitment	0.70**	0.62**	0.56**	0.65**
	*Remaining direct effects*				
γ_*k,1*_	Anxiety	0.16	–0.05	0.13	–0.23
γ_*k,2*_	Avoidance	−0.32*	–0.07	0.09	−0.35*
**Indirect effect on WtR**					
	*Through trust*				
$\beta_{k,1}^{T}*\gamma_{k,3}$	Anxiety	–0.09	−0.26 ° °	−0.19 ° °	–0.06
$\beta_{k,2}^{T}*\gamma_{k,3}$	Avoidance	–0.15	−0.38 ° °	−0.32 ° °	–0.11
	*Through satisfaction*				
$\beta_{k,1}^{S}*\gamma_{k,4}$	Anxiety	−0.37 ° °	−0.32 ° °	−0.29 ° °	−0.44 ° °
$\beta_{k,2}^{S}*\gamma_{k,4}$	Avoidance	−0.42 ° °	−0.33 ° °	−0.38 ° °	−0.51 ° °
	*Through commitment*				
$\beta_{k,1}^{C}*\gamma_{k,5}$	Anxiety	−0.20 ° °	−0.17 ° °	−0.12 ° °	−0.17 ° °
$\beta_{k,2}^{C}*\gamma_{k,5}$	Avoidance	−0.52 ° °	−0.45 ° °	−0.45 ° °	−0.49 ° °

*p < 0.05; **p < 0.01; °: 0 not contained in the 95% CI; °°: 0 not contained in the 99% CI.

All models show good explanatory power, with *R*^2^ values ranging between 0.42 (energy) and 0.55 (banking). Similar to the results reported in previous sections, while significance levels vary over the industries, the general patterns are (directionally) the same.

In the insurance and energy industries, effects of both RS anxiety and RS avoidance on WtR are fully mediated through the significant effects of both dimensions on trust, satisfaction, and commitment, which in turn affect respondents’ WtR. While banking and telecom show similar directional patterns, both industries show an insignificant effect of trust on respondents’ WtR. Moreover, while we find a situation of full mediation for RS anxiety, we observe a significant remaining direct negative effect of RS avoidance on WtR, implying a situation of partial mediation for this dimension in these industries. Overall, the preponderance of significant indirect effects (and absence of direct effects) of RS anxiety and RS avoidance through trust, satisfaction, and commitment on WtR provides support for H5a.

Both RS anxiety and RS avoidance show consistently negative effects on trust (supporting H2a and H2b). Effects of the latter, however, are 1.5–2 times stronger than those of the former with individual-industry differences all being significant (*p* < 0.01, for all industries), providing support for H2c. In a second step, trust has a positive effect on WtR, but this effect is only significant in the insurance and energy industries. As a consequence, indirect effects of both dimensions through trust are only present in those industries.

Similar to trust, respondents’ satisfaction is strongly depending on their RS anxiety and avoidance (supporting H3a and H3b). Although the difference in effect sizes of both dimensions is much smaller compared to the difference in effects on trust, here as well, RS avoidance shows a stronger impact compared to RS anxiety. While individual-industry differences are only significant in the energy industry (*p* < 0.05), the Added Z meta-analytic test confirms the significance of the pattern across the different industries (*p* < 0.05), thus providing support for H3c. Higher RS avoidance scores will thus have a stronger negative impact on satisfaction than similar higher RS anxiety scores. Resulting indirect effects on WtR through satisfaction are fully in line with this observation.

While effects of RS anxiety and RS avoidance on commitment show similar patterns as the ones described above (supporting H4a and H4b), the difference in effect sizes becomes much more outspoken, with RS avoidance showing effects which are 3–4 time stronger than those of RS anxiety, and individual-industry differences all being significant (*p* < 0.01, for all industries). Scoring high on RS avoidance will clearly have a much stronger detrimental impact on commitment compared to scoring high on RS anxiety, which is in line with H4c. This difference is also reflected in the resulting indirect effects on WtR.

### Robustness Checks

To confirm the validity of our findings, we applied several alternative model specifications. A first alternative allows for an interaction effect of anxiety and avoidance, where both attachment style dimensions are allowed to reinforce or mitigate one another in their effects on WtR. Except for the banking industry’s RS model, all models showed a slightly worse fit as expressed by an increased Bayesian Information criterion (BIC). Moreover, these models all suffered from severe multicollinearity, thus considerably reducing their usefulness^4^.

A second alternative uses the RS and G attachment style *types* (see section “Attachment Style Types”) instead of the RS and G attachment style *dimensions*. It thereby takes the secure type as reference type. The replacement of the continuous dimension variables by dichotomous type variables results in a considerable loss of information. This is also reflected in both reduced *R*^2^ values (ranging between 0.12 and 0.16 for RS attachment style types, and between 0.00 and 0.02 for G attachment style types) and increased (worse) BIC values compared to our focal model. Detailed results of the models using these alternative specifications are provided in the Supplementary Appendix.

An alternative conceptual reasoning could consider customers’ RS and G attachment styles not as antecedents of trust, commitment and satisfaction, but as moderators of their effects on WtR. We implemented such model specification as well, but could not find support for aforementioned moderating effects. Results of these analyses are available with the authors upon request.

## Attachment Style Types

### Defining Attachment Style Types

While this research analyses the effects of the attachment style *dimensions* anxiety and avoidance on customers’ trust in, satisfaction with, and commitment to the firm, ultimately resulting in a certain level of WtR, existing literature on attachment styles often uses these attachment style dimensions to define so-called attachment style *types*, depending on whether people score low/high on the attachment style dimensions (e.g., Van IJzendoorn et al., 2006). We therefore translate our findings (based on the dimensions) into insights for the different attachment style types proposed in the existing literature.

Based on the scores on the RS and G attachment style dimensions described above, we define four different attachment style types (see e.g., Van IJzendoorn et al., 2006). As both anxiety and avoidance (both RS and G) are measured using five-point Likert scale items, we classify a respondent with an average score of 3.1 or higher (i.e., scoring above the scale midpoint) on the anxiety (avoidance) dimension as “high” while a respondent with an average score below 3.1 (i.e., scoring below or equal to the scale midpoint) is considered “low” on that dimension. Subsequently, we combine the scores of the respondents on the two dimensions into a final RS and G attachment style type: Secure (low anxiety, low avoidance), Anxious (high anxiety, low avoidance), Avoidant (low anxiety, high avoidance), and Disorganized attachment (high anxiety, high avoidance) (see e.g., Van IJzendoorn et al., 2006). The order of the different styles as presented reflects the expected order of severity of deviation from the benchmark secure attachment style.

### Attachment Style Type Distribution

**FIGURE 2:**
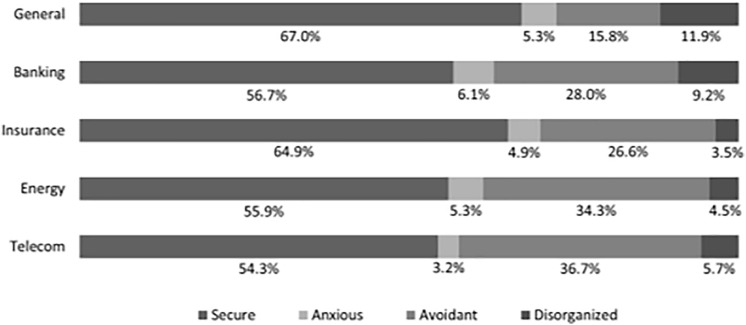
Distribution of general and relationship-specific attachment style types.

The distribution of the different attachment style types is depicted in Figure 2^5^. This figure shows that fewer people were secure in terms of RS attachment style in comparison with G attachment style. This difference can be explained by a different attachment target, namely other people for the G attachment style and a specific firm for the RS attachment style (Bartz and Lydon, 2004; Fraley et al., 2011). Another explanation is that an interpersonal relationship differs from a customer-firm relationship in interests (the latter which are compensatory attachment figures or attachment targets; e.g., Granqvist et al., 2010). While in an interpersonal relationship the interests are more likely to be similar, in a customer-firm relationship the customer and the firm have different, and possibly even opposing interests. The customer wants a certain service, while the firm wants to make a profit.

When comparing the RS attachment style types across industries, we also notice strong differences. People were most secure in the insurance industry and least in the telecom sector. This is the more interesting as research on RS attachment style so far has been limited to the insurance industry. Furthermore, more respondents were avoidant in the RS attachment style in the energy and telecom sector compared to the insurance and banking sector. Customers may care more about their financial services and want to have a stronger relationship with their bank or insurance company (hence activating different goals; Overall et al., 2003). The division of RS anxious and disorganized respondents was rather similar across the industries, with a notably higher percentage of disorganized respondents in the banking sector. These types of customers represent only a small part of the sample. Especially RS disorganized is an extreme attachment style type, so it is reasonable that only a small group of people has this attachment style type. These customers likely show this across industries and not just for one specific industry or firm.

### Attachment Style Types and WtR

Using the estimated model parameters for the RS attachment style dimensions, we can determine what the results would entail in terms of customers’ WtR and the impact of trust, satisfaction, and commitment for each of the four RS attachment style types (secure, anxious, avoidant, and disorganized), thereby taking the RS secure type as reference type. We thereby proceed in the following way:

1. For each of the four included industries, we first classify the respondents according to the procedure described above as one of the four RS types (secure, anxious, avoidant, disorganized) for that industry.
2. Subsequently, we use the estimated model parameters for RS anxiety and RS avoidance reported in Table 4, and multiply them with each respondent’s RS anxiety and RS avoidance scores to quantify the effect of the two RS attachment style dimensions on the respective outcome variables (NPS, trust, commitment, satisfaction) for that respondent in that particular industry.
3. For each of the four industries, we determine for each of the RS attachment style types the average score on these outcome variables across the respondents who are classified in the respective RS attachment style types.
4. We test whether these averages are (overall) significantly different by means of an ANOVA, and use the Dunnett’ C approach to test for significance of differences between the averages of the respective RS attachment style types.

Table 5 reports the differences in WtR for the RS anxious, avoidant, and disorganized types relative to the RS secure reference type. All values are relative to the 1–10 scale used to measure WtR. Across all industries a clear pattern emerges, with in order of magnitude anxious, avoidant, and disorganized types showing significantly lower WtR compared to the secure type. In line with what theory would predict, the difference is especially strong for the disorganized type that combines a high level of anxiety with a high level of avoidance. This type shows differences relative to the secure type that are 2–3 larger than those of the anxious and avoidant types. Moreover, while differences between the anxious and avoidant types are not significant in the insurance and energy industries, in all industries differences between the disorganized and any other RS attachment style type are always significant.

**TABLE 5:** Difference in WtR relative to RS secure customers.

	**Banking**	**Insurance**	**Energy**	**Telecom**
Anxious	−0.71 ^s,av,d^	−0.95 ^s,d^	−0.82 ^s,d^	−0.64 ^s,av,d^
Avoidant	−1.26 ^s,an,d^	−1.17 ^s,d^	−1.13 ^s,d^	−1.21 ^s,an,d^
Disorganized	−3.08 ^s,an,av^	−2.77 ^s,an,av^	−2.00 ^s,an,av^	−3.32 ^s,an,av^

*Letters in superscript indicate significant (*p* < 0.05) differences between the focal RS attachment style type and the secure (s), anxious (an), avoidant (av), or disorganized (d) RS attachment style type.*

The particular nature of RS disorganized customers also emerges from Table 6. In nearly every instance, this type of customers shows significantly lower trust, satisfaction, commitment, and ultimately WtR compared to the RS secure, anxious, and avoidant types. RS secure customers, in turn, in nearly every instance show significantly higher trust, satisfaction, commitment, and ultimately WtR compared to the RS anxious, avoidant, and disorganized types. While with regard to satisfaction, RS anxious and avoidant types resemble each other in some industries, they are clearly different when it comes to commitment and remaining direct effects on WtR. In those cases, RX anxious customers show stronger resemblance with RS secure customers.

**TABLE 6:** Difference on the mediation analysis aspects relative to RS secure customers.

		**Banking**	**Insurance**	**Energy**	**Telecom**
**Effect on trust**					
	Anxious	−0.33^s,*av*,d^	−0.44^s,d^	−0.35^s,d^	−0.20^s,*av*,d^
	Avoidant	−0.54^s,*an*,d^	−0.53^s,d^	−0.46^s,d^	−0.52^s,*an*,d^
	Disorganized	−1.37^s,*an*,*av*^	−1.28^s,*an*,*av*^	−0.84^s,*an*,*av*^	−1.32^s,*an*,*av*^
**Effect on satisfaction**					
	Anxious	−0.43^s,d^	−0.53^s,*av*,d^	−0.44^s,d^	−0.43^s,d^
	Avoidant	−0.44^s,d^	−0.39^s,*an*,d^	−0.44^s,d^	−0.42^s,d^
	Disorganized	−1.34^s,*an*,*av*^	−1.22^s,*an*,*av*^	−0.92^s,*an*,*av*^	−1.41^s,*an*,*av*^
**Effect on commitment**					
	Anxious	−0.21^s,*av*,d^	−0.29^s,*av*,d^	−0.18^*av*,d^	−0.01^*av*,d^
	Avoidant	−0.69^s,*an*,d^	−0.74^s,*an*,d^	−0.81^s,*an*^	−0.68^s,*an*,d^
	Disorganized	−1.43^s,*an*,*av*^	−1.32^s,*an*,*av*^	−0.99^s,*an*^	−1.44^s,*an*,*av*^
**Direct effect on WtR**					
	*Remaining direct effects*				
	Anxious	0.05^*av*,d^	0.00	0.00	0.18^*av*,d^
	Avoidant	−0.31^s,*an*,d^	0.00	0.00	−0.34^s,*an*,d^
	Disorganized	−0.43^s,*an*,*av*^	0.00	0.00	−0.50^s,*an*,*av*^
**Indirect effect on WtR**					
	*Through trust*				
	Anxious	0.00	−0.30^s,d^	−0.21^s,d^	0.00
	Avoidant	0.00	−0.36^s,d^	−0.29^s,d^	0.00
	Disorganized	0.00	−0.87^s,*an*,*av*^	−0.52^s,*an*,*av*^	0.00
	*Through satisfaction*				
	Anxious	−0.35^s,d^	−0.39^s,*av*,d^	−0.34^s,d^	−0.42^s,d^
	Avoidant	−0.36^s,d^	−0.29^s,*an*,d^	−0.33^s,d^	−0.39^s,d^
	Disorganized	−1.10^s,*an*,*av*^	−0.89^s,*an*,*av*^	−0.70^s,*an*,*av*^	−1.34^s,*an*,*av*^
	*Through commitment*				
	Anxious	−0.15^s,*av*,d^	−0.18^s,*av*,d^	−0.10^*av*,d^	−0.01^*av*,d^
	Avoidant	−0.48^s,*an*,d^	−0.46^s,*an*,d^	−0.45^s,*an*^	−0.44^s,*an*,d^
	Disorganized	−0.99^s,*an*,*av*^	−0.83^s,*an*,*av*^	−0.56^s,*an*^	−0.93^s,*an*,*av*^

*Letters in superscript indicate significant (*p* < 0.05) differences between the focal RS attachment style type and the secure (s), anxious (an), avoidant (av), or disorganized (d) RS attachment style type.*

## Discussion

### Summary of Findings

The goal of this paper was to provide an integrative perspective on customers’ RS and G attachment styles, customer-firm relationships, and customers’ WtR. More specifically, it engaged in an in-depth investigation on how customers’ RS and G attachment styles affect their WtR through the RS and G attachment styles’ effect on the customers’ experience with the firm as reflected in trust in, satisfaction with, and commitment to the firm. We conceived these three relationships dimensions separately because of their assumed differential relationships with attachment styles.

Our findings show that higher (relationship-specific) anxiety and especially higher avoidance significantly lower WtR the firm. In line with previous research, these RS attachment styles also show much better explanatory power compared to G attachment styles in explaining customers’ WtR, and this across all four included service industries. Note as well that the correlations between RS and G attachment style scores were low, thus adding to the need for using RS attachment scales in research on how customer experience and engage the relationship with firms. Note again that this finding conforms other work like Fraley et al. (2011) and partially Gruda and Kafetsios (2020) in different other contexts, the first in a social relationship context and the second in an organizational context (transference processes that occur during leadership interaction).

The RS attachment style dimensions anxiety and avoidance, in turn, showed significant negative effects on the relation dimensions of trust in, satisfaction with, and commitment to the firm, with negative effects being particularly strong for avoidance when it comes to its effect on trust and especially commitment. Customers with a higher level of RS anxiety, although showing a stronger fear for rejection (push), feel pulled to their attachment figures/targets (Vrticka and Vuilleumier, 2012). Customers with a higher level of RS avoidance, on the other hand, deliberately want to keep a larger distance, and want to commit less to the attachment figure.

Also in line with previous literature, all three relationship dimensions (trust, satisfaction, commitment) had a positive relation with the customers’ WtR, and eyeballing shows satisfaction to have the highest impact on WtR. This, in turn, shows the indirect effect of customers’ RS attachment style on their WtR through the effect on relationship dimensions. The customer relationship process model we proposed, besides including the indirect effect of the RS attachment style dimensions anxiety and avoidance on WtR through relationship dimensions, also allowed for direct effects of both RS attachment style dimensions on WtR. Only RS avoidance was found to have some direct effects in two industries which indicates, as Overall et al. (2003, p. 1491) argue, that attachment working models are activated depending on the current goals and past experience.

### Discussion

In our networked societies, customers’ evaluations of service experiences with firms and their resulting WtR behavior have become of ever more importance. Their recommendations first of all have a signaling function to other customers on the service performance by the firm. Secondly, referral value is included in customer equity valuation which is advocated to be included in financial reporting as a signal to investors on the value of the firm. Finally, managers steer on WtR based measures like NPS, and get evaluated and remunerated based on their scores. This study, however, shows that the signaling value of WtR measures is severely affected by a systematic bias in WtR based on the RS attachment style of the customer. It thus contributes to a better understanding of the relatively low predictive value of such measures like the NPS. While originally framed as the only metric one should know and use (Reichheld, 2003), more and more research has shown that it does not perform better than other metrics (e.g., Keiningham et al., 2007a, b). Our research shows that more insecure RS attachment styles, expressed by higher anxiety and/or avoidance, significantly lower customers’ trust, satisfaction, and commitment, which in turn will result in significantly lower WtR. At the same time, Mende et al. (2013) show that these relationship dimensions which they gather under the term relationship quality have no actual impact on loyalty behavior over time. This discrepancy between WtR and actual loyalty may hence be at the base of the low predictive power of WtR based measures.

This study also establishes a clear ranking in the severity of effects linked to the different RS attachment style dimensions. Customers with higher levels of only RS anxiety show the strongest resemblance with secure attached customers (the default, showing low levels of both RS anxiety and avoidance). Customers with higher levels of only RS avoidance, in turn, are more different from the latter. They are harder to please, because at the same time they expect the firm to stay at a distance yet remain highly sensitive to any signal of negative valence from the firm. They set the bar really high for firms, while at the same time not providing sufficient information for the firm to really cater to their needs in the most appropriate way. Thus, RS anxiety has considerably stronger biasing effects compared to RS avoidance.

Interestingly, the effect of customers’ RS attachment style on their WtR is nearly always fully indirect through its effect on trust, commitment, and satisfaction. Remaining direct effects could only be found for anxiety in some industries, but not all. As a consequence, the systematic way in which customers’ RS attachment style affects or biases their WtR appears of a different nature than the process through which it affects their repurchase intentions and loyalty behavior as investigated by Mende et al. (2013). While they show direct effects of RS attachment style on repurchase intentions together with an effect of relationship quality comprising trust, satisfaction, and commitment – which was shown by Mende and Bolton (2011) to be affected by RS attachment style – thus providing indirect evidence for partial mediation, only direct effects of RS attachment style on actual loyalty behavior could be found, without any indirect effects through relationship quality.

Our results also show that effects of RS attachment style on trust, satisfaction, and commitment, while following similar patterns, are not consistent in strength and significance. Hence, our decision not to combine them into one relationship quality construct (as Mende et al., 2013) appears warranted, as it provides us with more fine-grained insights in the process behind the bias in WtR based on RS attachment style. The usage of RS attachment styles also appears warranted, given much better explanatory power compared to G attachment styles. As such, it confirms that individuals’ RS attachment style can deviate from their G attachment style (e.g., Mikulincer and Shaver, 2007) and that such RS attachment style has much better predictive power (Klohnen et al., 2005; Fraley et al., 2011). In addition, it corroborates that such RS attachment style not only matters in interpersonal relations but also in relations to abstract entities like firms which thus function as attachment targets (e.g., Mende and Bolton, 2011; Mende et al., 2013).

### Managerial Implications

Responding to our findings on attachment styles, one manager told the authors during a meeting on the role of attachment styles in marketing that he “*now understood why some customers do not get any satisfaction*” and subsequently concluded that “*from now on [he] would not take negative evaluations by a segment of customers too personally*.” While understandable, this conclusion would be a limiting, perhaps even myopic interpretation of our findings that ignores other relevant implications for managers. Our research shows a systematic bias in WtR based measures of firms’ service performance, with both external and internal consequences. Systematic underestimation of the true service performance may lead to lower attractiveness to potential customers, lower attractiveness to investors, and lower rewards for employees when evaluated on such measures, possibly resulting in loss of motivation and true loss in service performance. In other terms, actual service performance declining is an indirect consequence of the under-rewarding of the actual service performance.

As a first step to avoid such infernal downward spiral, firms may be cautious in steering and evaluating their staff purely on the firms’ WtR based NPS performance, both at the individual customer level (as an absolute measure to predict future loyalty) and at the aggregate firm level (as an indicator of, e.g., future growth). As such, the claim by Reichheld (2003) that NPS should be “the one number you need to grow” appears overly optimistic, and managers are advised to combine consumer feedback metrics, taking into account possible biases due to personality traits of their customers. This holistic approach should result in a more accurate basis to evaluate staff, thus ensuring a more correct rewarding of their efforts and hence a more likely sustained motivation to provide good service to customers.

In a second step, firms may want to use these insights to obtain a better alignment of their customers’ WtR with the actual service performance. As a starting point, firms can survey their customers upfront on their customer-firm relationship preferences. Many firms already apply such upfront surveys. Adding RS attachment scales that focus on anxiety and avoidance will provide firms with the most relevant and directly applicable insights for that industry. It will also allow them to subsequently segment their customers into, for example, secure, anxious, avoidant, and disorganized customers. This, in turn, should allow them to deal with the specificities of these types of customers in the most productive way by not only adjusting their communication, but also by more intelligent use of existing information to better sense what the customers are looking for.

Customers with low levels of both RS avoidance and RS anxiety – so-called RS secure attached customers – are the customers that have the most “normal” relationships with the firm, and the most reliable WtR scores. Any offer aimed at this segment is likely to result in reliable increases in WtR: for these customers, WtR is the best reflection of the service experience. As such, these customers are the most likely to become true relationship-customers, showing high trust, satisfaction and commitment, resulting in considerably higher WtR.

Customers with higher levels of only RX anxiety are more afraid of being left alone by the service provider, of not being able to count on its services. Reassuring them by, for example, giving them testimonials of other customers on the great service by and contacts with the firm, or information on the steady service performance and reliability of the firm could still add some value. However, overall, firms may decide not to worry too much about this segment, and treat them similarly to the secure customers.

Customers with higher levels of only RS avoidance, on the other hand, may require a vastly different approach. The higher the level of RS avoidance, the more these customers want to keep the firm at a distance while at the same time they are more sensitive to errors made by the service firms. One could expect that the outcome of this two-sided behavior reflected in the service they receive is suboptimal in their eyes (negative expectancy-disconfirmation), leading to lower trust, satisfaction, commitment, and WtR. Hence, better sensing of their needs through more active data-mining could enable firms to develop better tailored offers to these customers, while at the same time respecting their need for distance. This, in turn, should provide these customers with a better experience, ultimately leading to higher and more accurate WtR as outcome of the service experience. The higher the level of RS avoidance, however, the more likely these customers are to remain arms-length “transactional” customers: they sign the contract, use the service, pay their bills and for the rest want to be left alone. Nevertheless, a happy arms-length customer can still be of higher value to the company than customers who defect because they feel the contact is too close. Moreover, such happy arms-length customers may be more inclined to recommend the firm that respects their desire to stay at arms-length, while receiving the desired service based upon intelligent data-mining techniques.

The higher customers score simultaneously on both RS anxiety and RS avoidance, the harder it becomes to serve them, thus causing the most frustration among managers. While data-mining applications may still provide these customers with a longed-for service experience, they also at the same time want to keep a greater distance and need to be comforted and reassured more, two desires which are clearly hard to reconcile. Question then becomes whether any success can be achieved in trying to serve these customers in such a way that they feel real trust in, satisfaction with, and commitment to the firm, and ultimately will be willing to recommend the firm. Divesting strategies may then become an option for these customers.

### Limitations

Our research, while providing better insights in how customers appraise their relationships with firms and engage in WtR accordingly, also shows some limitations. First, we did not study how customers relate to the firm’s salesperson as opposed to the firm as such, because many customers of the four industries included in this study use the Internet to connect with their service providers. As such, no interpersonal contact traits were included in our work, while they could be more relevant in other industries. Second, while this study focused on effects of RS attachment styles on WtR, other customer feedback metrics may also be affected by these personality traits. Combining feedback metrics to evaluate firms’ service performance as advocated by for example De Haan et al. (2015) hence calls for a further investigation of the existence and strength of such effects. Third, for reasons of consistency and to foster comparability with extant research, this research has used well-established scales prevalent in the relevant extant literature. Some of these scales, however, have relatively few items or even just one item. While some argue that single-item scales can be as good a multi-item scales (e.g., Rossiter, 2002), other researchers state that the usage of scales with few items could be dangerous as predictive validity could be variable across constructs (e.g., Diamantopoulos et al., 2012). Future research could repeat our analyses with new scales based on more items. Fourth, while our models show good explanatory value in terms of *R*^2^, we acknowledge that many other factors could affect customers WtR above and beyond the factors included in our model. Fifth, and linked to previous point, we could have included other personality-trait related variables in order to better comprehend what the RS and G attachment style scales actually mean. For example, do customers that show higher levels of RS and G anxiety really experience anxiety when dealing with firms? Sixth, we studied the role of attachment styles in a B2C environment only, consisting of four different industries. Extending the research to other B2C industries and B2B markets, with new and larger samples, could shed light on the extent to which our findings also hold in such settings. Finally, the concept of WtR as such only covers the positive referral facet in an explicit way. It does not cover the extent to which customers may be likely to criticize the firm. This is not without importance, as not recommending does not yet imply denouncing. Investigating this negative referral facet (“willingness-to-denounce”) as a complement to traditional WtR, and the extent to which it is driven by customers’ RS and G attachment styles, could provide valuable insights on the extent to which this negative facet is more/less reliable as a customer feedback metric.

## Data Availability Statement

The datasets generated for this study are available on request to the corresponding author.

## Ethics Statement

Ethical review and approval was not required for the study on human participants in accordance with the local legislation and institutional requirements. The patients/participants provided their written informed consent to have their data used for scientific research purposes.

## Author Contributions

WV developed hypotheses and wrote the manuscript. MG managed the project, computed the data, and wrote the manuscript. LH managed the project, developed the hypotheses, collected the data, and wrote up research results. JB managed the project. LT managed the data collection process.

## Conflict of Interest

LH and LT were employed by company MetrixLab. The remaining authors declare that the research was conducted in the absence of any commercial or financial relationships that could be construed as a potential conflict of interest.

## Footnotes

1 As this research is based on secondary data after informed consent, no formal ethical approval from the Research Ethics Boards from the involved universities was sought.

2 Subsequent analyses are done on a per-industry basis. Our approach is warranted given (a) that a same respondent can have a considerably different RS attachment style depending on the industry; (b) that the included industries are considerably different; (c) that the random assignment of respondents to two industries ensures absence of systematic industry combinations (i.e., respondents being systematically assigned to the same combination of two industries), thus avoiding systematic correlations between industries.

3 De Haan et al. (2015) refer to this question as the “untransformed NPS value.” Also note that this scale is not a multi item scale.

4 Past literature often suggested to mean-center the variables in order to reduce multicollinearity. This approach, however, has been shown not to be effective in doing so (Echambadi and Hess, 2007).

5 The reported results are in line with the findings by Van IJzendoorn et al. (2006), providing face validity for our classification.
