# Supplementary material for: Highly Recommended? How Relation-Specific Attachment Styles Bias Customers Willingness to Recommend
Source: Front Psychol. 2020 Jun 10;11:1311. doi: 10.3389/fpsyg.2020.01311 (PMC7298734; doi:10.3389/fpsyg.2020.01311)
Supplement: Supplementary file 1 [file Presentation_1.pdf]

Highly Recommended? How Relationship-Specific Attachment Styles Bias Customers' Willingness to Recommend

WEB APPENDIX

Table WA1. Overview of Scales

Variable	Question	Source
**General Attachment Style Dimensions**	*Anxiety*<br>I worry that others won't care about me as much as I care about them<br>My desire to be very close to others sometimes scares them away<br>I need a lot of reassurance that I am loved by others around me<br>I worry about being neglected or ignored by others<br>I get frustrated if others are not available when I need them<br>I feel a certain amount of anxiety in my relationships with others<br><br>*Avoidance*<br>I want to get close to others, but I keep pulling back<br>I am nervous when other people get too close to me<br>I try to avoid getting too close to others<br>I try to maintain a certain amount of distance between myself and others<br>I am very self-reliant in my dealings with others	Verbeke, Bagozzi, & van den Berg (2013)
**Relationship-specific Attachment Style Dimensions**	*Anxiety*<br>I worry that [firm] will abandon me as a customer<br>[Firm] changes how [it/she/he] treats me for no apparent reason<br>I worry that [firm] doesn't really like me as a customer<br>I worry that [firm] doesn't care about me as much as I care about [firm]<br><br>*Avoidance*<br>It is a comfortable feeling to depend on [firm]<br>I am comfortable having a close relationship with [firm]<br>It's easy for me to feel warm and friendly toward [firm]<br>It helps to turn to [firm] in times of need	Mende & Bolton, (2011);<br>Mende, Bolton, & Bitner (2013)
**Trust**	Firm/agent is trustworthy<br>Firm/agent keeps promises<br>Firm/agent is truly concerned about my welfare	Doney & Cannon (1997)
**Satisfaction**	How satisfied are you with [firm]?	Aaker, Foumier, & Brasel (2004)
**Commitment**	I enjoy being a customer of firm/agent<br>I have positive feelings about firm/agent<br>I feel attached to firm/agent	Coulter, Price, & Feick (2003);<br>Gruen, Summers, & Acito (2000)
**NPS**	How likely is it that you would recommend [firm] to a friend or colleague?	Reichheld (2003)

Table WA2. Bivariate Correlations between Constructs for the Banking Industry

	G Anxiety	G Avoidance	RS Anxiety	RS Avoidance	Trust	Satisfaction	Commitment	WtR
G Anxiety	1.00							
G Avoidance	0.57 **	1.00						
RS Anxiety	0.25 **	0.09	1.00					
RS Avoidance	-0.04	0.02	0.23 **	1.00				
Trust	-0.15 **	-0.11 *	-0.51 **	-0.65 **	1.00			
Satisfaction	-0.13 **	-0.06	-0.51 **	-0.52 **	0.73 **	1.00		
Commitment	-0.05	0.00	-0.40 **	-0.67 **	0.82 **	0.69 **	1.00	
WtR	-0.04	-0.03	-0.41 **	-0.54 **	0.66 **	0.67 **	0.67 **	1.00

*p < 0.05; ** p < 0.01

Table WA3. Bivariate Correlations between Constructs for the Insurance Industry

	G Anxiety	G Avoidance	RS Anxiety	RS Avoidance	Trust	Satisfaction	Commitment	WtR
G Anxiety	1.00							
G Avoidance	0.57 **	1.00						
RS Anxiety	0.25 **	0.17 **	1.00					
RS Avoidance	0.02	0.14 **	0.01	1.00				
Trust	-0.17 **	-0.23 **	-0.44 **	-0.60 **	1.00			
Satisfaction	-0.09	-0.12 *	-0.45 **	-0.43 **	0.68 **	1.00		
Commitment	-0.09	-0.19 **	-0.28 **	-0.66 **	0.76 **	0.60 **	1.00	
WtR	-0.08	-0.19 **	-0.32 **	-0.45 **	0.63 **	0.61 **	0.60 **	1.00

* $p < 0.05$; ** $p < 0.01$

Table WA4. Bivariate Correlations between Constructs for the Energy Industry

	G Anxiety	G Avoidance	RS Anxiety	RS Avoidance	Trust	Satisfaction	Commitment	WtR
G Anxiety	1.00							
G Avoidance	0.57 **	1.00						
RS Anxiety	0.32 **	0.23 **	1.00					
RS Avoidance	-0.13 *	-0.02	-0.10	1.00				
Trust	-0.10	-0.12 **	-0.32 **	-0.55 **	1.00			
Satisfaction	-0.14 **	-0.12 *	-0.34 **	-0.44 **	0.67 **	1.00		
Commitment	0.05	-0.00	-0.13 **	-0.70 **	0.73 **	0.60 **	1.00	
WtR	0.01	-0.04	-0.23 **	-0.41 **	0.57 **	0.56 **	0.57 **	1.00

* $p < 0.05$; ** $p < 0.01$

Table WA5. Bivariate Correlations between Constructs for the Telecom Industry

	G Anxiety	G Avoidance	RS Anxiety	RS Avoidance	Trust	Satisfaction	Commitment	WtR
G Anxiety	1.00							
G Avoidance	0.57 **	1.00						
RS Anxiety	0.27 **	0.23 **	1.00					
RS Avoidance	-0.13 *	-0.08	-0.00	1.00				
Trust	-0.03	-0.08	-0.35 **	-0.60 **	1.00			
Satisfaction	-0.09	-0.07	-0.41 **	-0.43 **	0.72 **	1.00		
Commitment	-0.02	0.00	-0.24 **	-0.61 **	0.78 **	0.66 **	1.00	
WtR	-0.04	-0.05	-0.32 **	-0.46 **	0.62 **	0.63 **	0.67 **	1.00

* $p < 0.05$; ** $p < 0.01$

Table WA6. Alternative Model Specification 1: Effects on WtR

	Banking	Insurance	Energy	Telecom
Relationship-Specific (RS)Attachment Style				
Constant	10.63**	10.86**	10.48**	11.67**
Anxiety	0.15	-0.17	0.11	-0.30
Avoidance	-0.71**	-0.85**	-0.72**	-1.00**
Anx*Avoidance	-2.79**	-0.20	-0.27	-0.19
Age	-0.00	0.01	0.00	0.00
Gender	-0.07	-0.14	-0.09	0.27
R^2	0.39	0.30	0.25	0.32
F-value	52.72**	31.43**	25.65**	36.46**
Max VIF	19.87	18.22	17.88	14.87
BIC	3.908	3.910	4.014	4.077
General (G) Attachment Style				
Constant	9.89**	8.91**	8.78**	8.75**
Anxiety	-1.28*	-0.45	-0.78	-0.70
Avoidance	-1.00*	-1.01*	-0.95*	-0.71
Anx*Avoidance	0.40*	0.20	0.33*	0.23
Age	-0.00	0.01	0.00	0.00
Gender	-0.07	-0.23	-0.15	0.15
R^2	0.02	0.05	0.02	0.01
F-value	1.35	3.59**	1.28	0.76
Max VIF	33.27	30.08	30.68	27.37
BIC	4.382	4.222	4.281	4.446

* $p < 0.05$, **: $p < 0.01$

Table WA7. Alternative Model Specification 2: Effects on WtR

	**Banking**	**Insurance**	**Energy**	**Telecom**
Relationship-Specific (RS)Attachment Style				
Constant	7.19**	6.65**	7.10**	6.99**
Anxious	0.04	-0.86	-0.38	-0.76
Avoidant	-1.00**	-1.22**	-0.99**	-0.90**
Disorganized	-2.79**	-2.48**	-2.88**	-3.38**
Age	-0.00	0.01*	0.00	0.00
Gender	-0.07	-0.12	-0.17	0.25
R^2	0.16	0.13	0.12	0.15
F-value	16.25**	10.79**	11.07**	13.52**
BIC	4.220	4.132	4.165	4.298
General (G) Attachment Style				
Constant	6.69**	6.37**	6.64**	6.73**
Anxious	-0.43	-0.00	-0.15	-0.19
Avoidant	0.05	-0.45	-0.13	-0.41
Disorganized	0.34	0.06	0.06	-0.12
Age	-0.00	0.01	0.00	0.00
Gender	-0.04	-0.19	-0.14	0.18
R^2	0.01	0.02	0.00	0.01
F-value	0.51	1.53	0.26	0.47
BIC	4.392	4.250	4.294	4.450

**p* < 0.05, ** *p* < 0.01
